# Supplementary material for: Recent PELE Developments and Applications in Drug Discovery Campaigns
Source: Int J Mol Sci. 2022 Dec 17;23(24):16090. doi: 10.3390/ijms232416090 (PMC9788188; doi:10.3390/ijms232416090)
Supplement: Supplementary file 1 [file ijms-23-16090-s001.zip › ijms-2075176-supplementary.pdf]

International Journal of *Molecular Sciences*

MDPI

Review

Supplementary Information: Recent PELE Developments and Applications in Drug Discovery Campaigns

Ignasi Puch-Giner [1], Alexis Molina [2], Martí Municoy [1,2], Carles Pérez [2] and Victor Guallar [1,2,*]

1 Barcelona Supercomputing Center, Plaça d'Eusebi Güell, 1-3, 08034 Barcelona, Spain
2 Nostrum Biodiscovery S.L., Av. de Josep Tarradellas, 8-10, 3-2, 08029 Barcelona, Spain
* Correspondence: victor.guallar@bsc.es

1. Methods

1.1. System Preparation

For each system, crystals with missing atoms in side chains or missing loops were corrected with Maestro and its 3D Builder [48]. To determine the connectivity and the protonation states we used the Protein Preparation Wizard from Schrödinger [49] as well as PROPKA [50] at the pH at which the crystallization of the complex had been done.

1.2. AquaPELE and fragPELE Simulation Protocol

OPLS2005 [13] force field was used to parameterize protein residues for both aquaPELE simulations and Frag + aquaPELE simulations. We used the SGB solvent model [51] in the implicit/explicit solvent protocol in aquaPELE simulations. We set the aquaPELE simulations to explore the possible ligand conformations and hydrated sites within the receptor's binding site. We performed 47 parallel simulations of 400 Monte Carlo steps (18.800 total steps). The fragment growth was implemented in 10 growing epochs of 6 Monte Carlo steps each. The final sampling simulation consisted of 400 Monte Carlo steps, comparable to the scaffold-alone simulation.

1.3. Scoring Metrics for Energetic Benchmark

- P5: Average binding energy of 5th percentile of all the simulation.
- POP5: Average binding energy of 5th percentile of all snapshots belonging to the most populated ligand cluster.
- P25: Average binding energy of 25th percentile of all the simulation.
- POP25: Average binding energy of 25th percentile of all snapshots belonging to the most populated ligand cluster.
- MBE: Average binding energy of all the simulation.
- POPMBE: Average binding energy of all snapshots belonging to the most populated ligand cluster.

check for updates

Citation: Puch-Giner, I.; Molina, A.; Municoy, M.; Pérez, C.; Guallar, V. Recent PELE Developments and Applications in Drug Discovery Campaigns. *Int. J. Mol. Sci.* **2022**, *23*, 16090. https://doi.org/10.3390/ijms232416090

Received: 18 November 2022
Accepted: 13 December 2022
Published: 17 December 2022

Publisher's Note: MDPI stays neutral with regard to jurisdictional claims in published maps and institutional affiliations.

Copyright: © 2022 by the authors. Licensee MDPI, Basel, Switzerland. This article is an open access article distributed under the terms and conditions of the Creative Commons Attribution (CC BY) license (https://creativecommons.org/licenses/by/4.0/).

2. Images and Tables

Table S1. Structural information of all fixed water molecules for each system according to the labelling of the indicated PDB.

<table>
<tr><th colspan="2">Systems</th><th>PDB scaffold</th><th>Fixed Waters IDs</th></tr>
<tr><td colspan="2">HSP90 (1)</td><td>3RLQ</td><td>237,258,313,243,231</td></tr>
<tr><td colspan="2">HSP90 (2)</td><td>2XAB</td><td>2118,2155</td></tr>
<tr><td colspan="2">BRD4</td><td>5I80</td><td>352,364</td></tr>
<tr><td colspan="2">TAF1</td><td>5I29</td><td>1832,1882</td></tr>
<tr><td colspan="2">SiaP WT</td><td>2V4C</td><td>2198,2016,2088,2343,
2253,2037,2228,2251,2110,
2109,2226,2344,2345</td></tr>
<tr><td colspan="2">CHK1</td><td>2C3L</td><td>-</td></tr>
<tr><td rowspan="2">Control</td><td>HSP90 (1)</td><td>3RLQ</td><td>237,258,313,243,231</td></tr>
<tr><td>HSP90(3)</td><td>-</td><td>261,297,372,311,
336,292,229,274,310,
370,230,240,244</td></tr>
</table>

Table S2. Set of grown fragments from the original scaffold L1 (Supplementary Figure S2). Their respective energetic toll has been calculated with the experimental K_i with $\Delta\Delta G = k_B T \ln(K_i)$.

Compound	**L1**			$\Delta\Delta G$ (kcal mol^{-1})
	R1	R2	R3	
Kung-4	OCH_3	H	H	-6.95
Kung-5	OCH_3	Cl	H	-7.59
Kung-6	Cl	Cl	H	-8.80
Kung-7	Cl	Cl	OCH_3	-9.34

Table S3. Set of grown fragments from the original scaffold L2 (Supplementary Figure S2). Their respective energetic toll has been calculated with the experimental K_i with $\Delta\Delta G = k_B T \ln(K_i)$.

Compound	L2		$\Delta\Delta G$ (kcal mol^{-1})
	R1	R2	
Kung-10	$COCH_3$	H	-9.13
Kung-11	F	H	-9.13
Kung-12	Cl	H	-9.13
Kung-13	CN	H	-10.08
Kung-14	H	CH_3	-7.77
Kung-15	Cl	CH_3	-9.54
Kung-16	CN	CH_3	-10.25
Kung-17	CN	CH_2CH_3	-10.66

Table S4. Set of grown fragments from the original scaffold L3 (Supplementary Figure S2). Their respective energetic toll has been calculated with the experimental K_i with $\Delta\Delta G = k_B T \ln(K_i)$.

Compound	L3				$\Delta\Delta G$ (kcal mol^{-1})
	R1	R2	R3	R4	
Woodhead-2	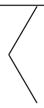	H	H	H	-12.63
Woodhead-5	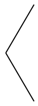	H	H	CH_3	-10.66
Woodhead-6	CH_2CH_3	H	H	H	-11.85
Woodhead-7	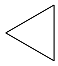	H	H	H	-11.44
Woodhead-8	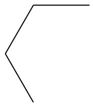	H	H	H	-11.61
Woodhead-9	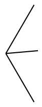	H	H	H	-12.26
Woodhead-10	Cl	H	H	H	-11.44
Woodhead-11	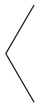	H	F	H	ND
Woodhead-12	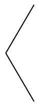	F	H	H	ND

Table S5. Correlation between the experimental data in the literature and predicted data depending on the protocol (W, FW, NW) and the scoring method for the first series of congeneric ligands (L1). These correlations were calculated with four data points corresponding to each fragment-grown compound in Supplementary Table S2.

Serie 1 (L1)

Scoring method	r(W)	r(FW)	r(NW)
P5	0.98	0.96	0.71
P25	0.97	0.93	0.72
MBE	0.92	0.91	0.75
POP5	0.99	0.96	0.76
POP25	0.97	0.93	0.78
POPMBE	0.92	0.91	0.76

Table S6. Correlation between the experimental data in the literature and predicted data depending on the protocol (W, FW, NW) and the scoring method. These correlations were calculated with eight data points corresponding to each objective compound in Supplementary Table S3.

Serie 2 (L2)

Scoring method	r(W)	r(FW)	r(NW)
P5	0.78	0.75	0.65
P25	0.72	0.83	0.58
MBE	0.78	0.84	0.80
POP5	0.76	0.61	0.63
POP25	0.73	0.63	0.50
POPMBE	0.73	0.61	0.48

Table S7. Correlation between the experimental data in the literature and predicted data depending on the protocol (W, FW, NW) and the scoring method. These correlations were calculated with seven data points corresponding to the first seven objective compounds in Supplementary Table S4.

Serie 3 (L3)

Scoring method	r(W)	r(FW)	r(NW)
P5	0.58	0.39	0.24
P25	0.46	0.29	0.31
MBE	0.48	0.30	0.27
POP5	0.58	0.32	0.31
POP25	0.44	0.20	0.31
POPMBE	0.50	0.16	0.22

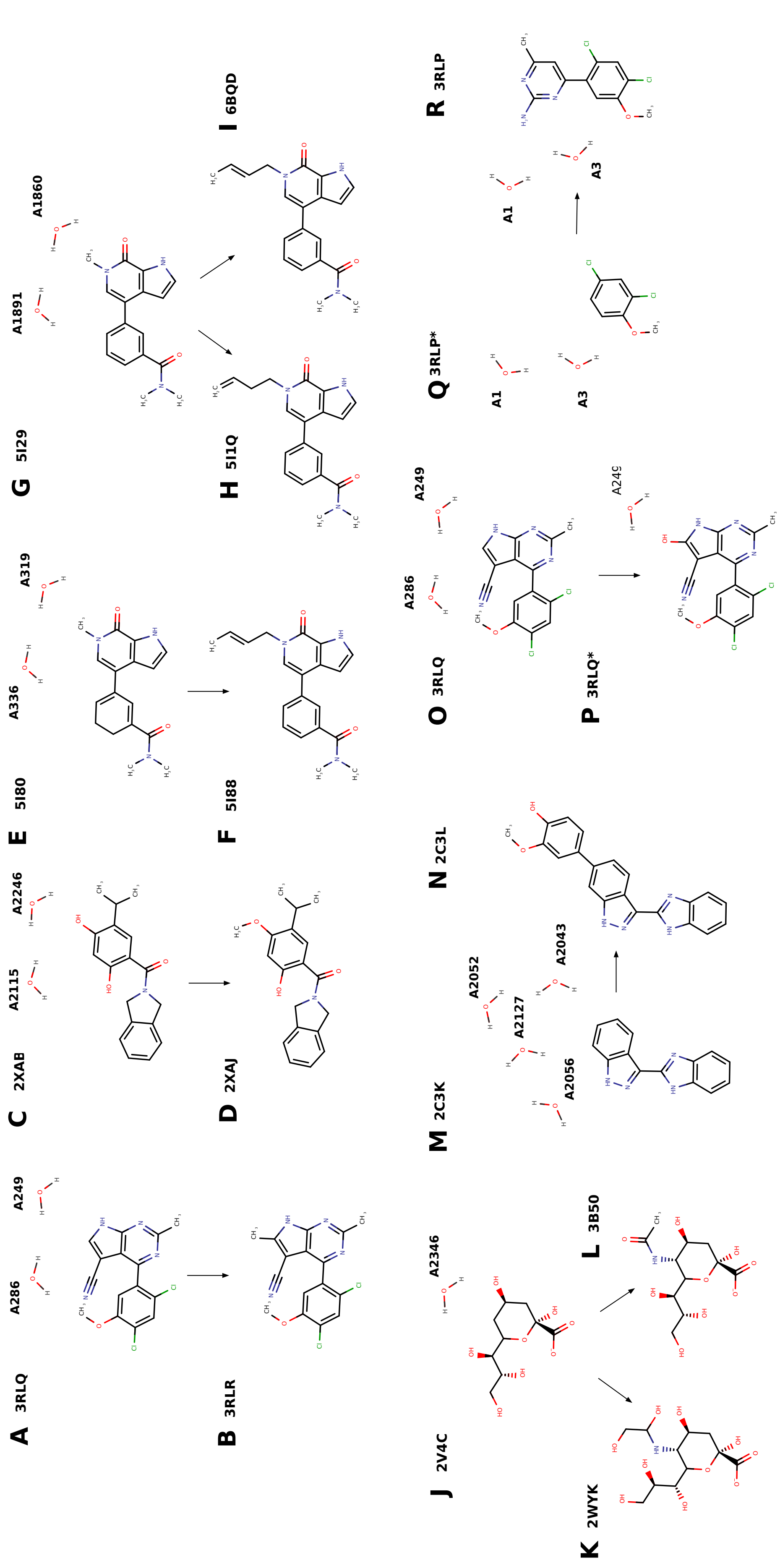

Figure S1. Set of systems considered in the structural study benchmark. PDB codes are indicated along with the labelling of each ligand structure. It has binders to the HSP90 (panels A-D and O-R), BRD4 (E and F), TAF1 (G-I), SiaP WT (J-L) and CHK1 (M and N) [43–47] Water molecules have been labelled according to the indicated PDB. *Structure that doesn't have a crystal but has been modified from the indicated PDB.

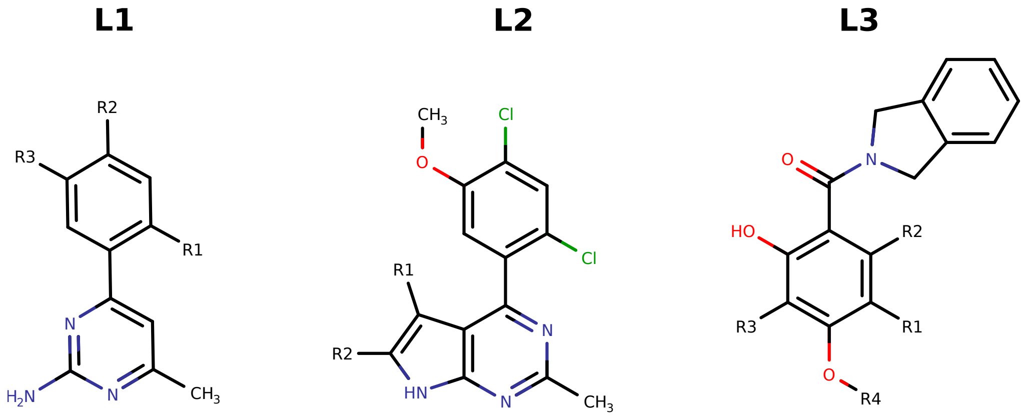

Figure S2. Set of initial scaffolds considered in the energetic study benchmark. These HSP90 inhibitors have not been crystallized in complex with the protein. R1, R2, R3 and R4 indicate regions where the fragments from the Supplementary Tables S2, S3 and S4 are going to be grown. Compounds and energetic data have been retrieved from the literature [43,44].
